# Can the cross-borders directive improve the quality of genetic testing in the future?

**DOI:** 10.1186/1750-1172-7-S2-A7

**Published:** 2012-11-22

**Authors:** Jean-Jacques Cassiman

**Affiliations:** 1 Center for Human genetics, KU Leuven, Leuven, Belgium

## 

A survey of a series of genetic centers in Europe [1] showed that today a substantial number of samples from patients are being sent abroad for DNA analysis to laboratories in Europe, the US or elsewhere in the world. 66% of the 233 laboratories, which replied to the questionnaire, received samples from other countries and 47% sent samples to other countries. In absolute numbers this would mean that about ¼ of all samples cross borders today. This traffic will only further increase when the European cross-border directive 2011/24 [2] will be activated. This directive states that “Member States shall bring into force the laws, regulations and administrative provisions necessary to comply with this Directive by 25 October 2013”. The focus of these regulations and provisions should be on the quality of the services wherever the patients or their samples are being examined [3]. The quality management of the facilities i.e. evidence that they follow international guidelines, e.g. those published in 2007 by the OECD [4] or are accredited under the ISO 15189; that appropriate pre- and post- testing counseling is guaranteed, particularly when whole genome or exome sequencing is considered; and that patient representatives are involved in the implementation of these processes, should become compulsory. The Direct to Consumer (DTC) testing facilities, available through internet, have forced geneticists to reconsider the way genetic services are provided. A positive aspect of this development is that issues, such as information for prospective consumers, counseling and support, consent, data protection, interpretation of results, and others have received much more attention than in the past. The EuroGentest Network of Excellence (NoE), supported by DG Research (2005 – 2010)and its successor as Coordination Action (2011-2013), together with the European Society of Human Genetics, have worked very hard on the improvement of the quality of the genetic services in Europe. Nevertheless, such efforts by ‘volunteers’, will not suffice once the new Directive is in place. To guarantee quality services to the consumers we will need harmonisation of the services, which can only be achieved at the European level through the existing facilities of the commission. There is a need for a regulatory framework for Quality Assurance (ISO accreditation), for test development and use, harmonised legal, regulatory and healthcare policies for pharmacogenetics as well as a the finalization of the revision of the IVD directive, preferably in harmony with the ‘Global Health Task Force’.
